# Rectal adenocarcinoma metastatic to the tonsil; PET-CT observations with pathological confirmation: A case report

**DOI:** 10.3892/ol.2013.1671

**Published:** 2013-11-08

**Authors:** JIAN-PING HE, SHUANG ZHANG, ZONG-GUO PANG, QIU LI

**Affiliations:** Cancer Center, State Key Laboratory of Biotherapy, West China Hospital, Sichuan University, Chengdu, Sichuan 610041, P.R. China

**Keywords:** tonsil, metastasis, rectal adenocarcinoma

## Abstract

Metastasis of rectal adenocarcinoma develops by lymphatic or hematogenous spread. The usual sites of metastasis from rectal adenocarcinoma include local and distant lymph nodes, the liver and the lungs. The current case report presents a unique case of a mass that was identified in the tonsil by positron emission tomography-computed tomography (PET-CT), indicating a metastasis from rectal adenocarcinoma. Metastatic tumor to the tonsil is extremely rare and to the best of our knowledge, no previous studies have reported a case of tonsil metastasis from rectal adenocarcinoma. PET-CT scanners represent an important evolution in technology that is helping to bring anatomical imaging togeother with functional imaging in cancer diagnosis and therapy. Written informed consent was obtained from the patient.

## Introduction

Rectal cancer is the common cause of cancer-related mortality in the United States, with ~40,290 new cases in 2012 (1). Rectal tumors spread locally and metastasize to distant sites. The liver is the most common site of metastasis, followed by the regional lymph nodes, lungs and bone (2). The most important prognostic factor in rectal adenocarcinoma is the stage of the tumor, which also indicates the therapeutic strategy. However, accurate detection of rectal carcinoma remains a diagnostic challenge. Although surgery is the most effective primary treatment for rectum carcinoma patients in early stage, it may not be suitable for patients with disseminated metastasis. Therefore, due to incorrect staging, certain patients with disseminated disease may be subjected to unnecessary surgery that may result in considerable morbidity (3). By detecting disease and predicting resectability, positron emission tomography-computed tomography (PET-CT) offers an efficient means to manage such patients. A PET-CT scanner combines PET and CT cameras, and is a new device with significant diagnostic potential. Compared with PET alone, PET-CT shows significant increase in the certainty of lesion localization and characterization of rectal adenocarcinoma (4).

## Case report

A 67-year-old male, diagnosed with stage III rectal adenocarcinoma, was admitted to West China Hospital (Chengdu, China) for abdominal rectal resection in May 2010. Postoperative pathological examination indicated mucosal adenocarcinoma, invading the whole intestinal wall and tumor cells were identified in 4/4 mesenteric lymph nodes.

**Figure 1:**
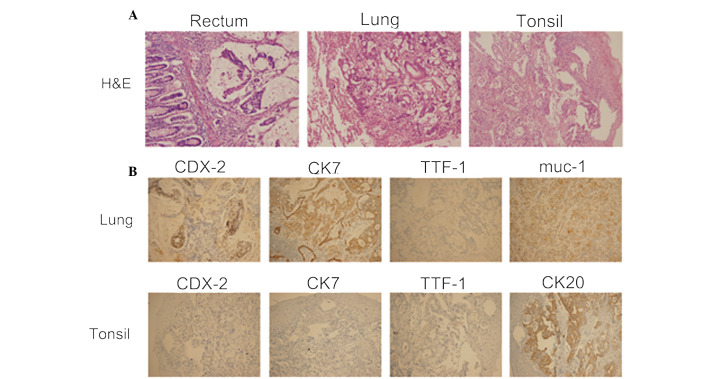
(A) Hematoxylin and eosin staining of the tumor cells in the rectum, lung and tonsil. (B) Immunohistochemical staining of the lung showed CDX-2(+), muc-1(+), CK7(+) and TTF-1(−) in the tumor cells. Immunohistochemical staining of the tonsil showed CDX-2(−), CK20(+), CK7(−) and TTF-1(−) in the tumor cells, confirming metastasis from colorectal adenocarcinoma (magnification, ×400). CDX-2, caudal type homeobox transcription factor 2; muc-1, mucin-1; CK7, cytokeratin 7; TTF-1, thyroid transcription factor-1.

The patient was treated by chemotherapy with oxaliplatin, calcium folinate and fluorouracil every 14 days for seven cycles. In March 2011, PET-CT revealed a left lower lobe nodule with mildly increased glucose metabolism. The largest standardized uptake value (SUV) was 2.38, which exhibited metastatic possibility. The pulmonary nodule was surgically removed and the postoperative pathological diagnosis was a poorly differentiated adenocarcinoma arising from primary rectal adenocarcinoma. The tumor was diagnosed as invading the visceral pleura and immunohistochemical staining showed caudal-type homeobox transcription factor 2 (CDX-2)(+), mucin-1(+), cytokeratin 7 (CK7)(+) and thyroid transcription factor-1 (TTF-1)(−) in the tumor cells (Fig. 1).

**Figure 2:**
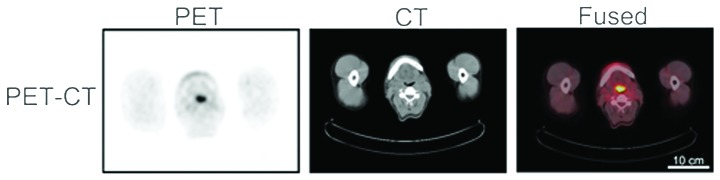
PET, CT and fused images. The tonsil was swollen and the largest standardized uptake value was 8.08 (scale bar, 10 cm). PET, positron emission tomography; CT, computed tomography.

Subsequently, the patient was treated by chemotherapy with 50 mg TS-1 twice daily. In November 2011, PET-CT revealed that the tonsil was swollen and the largest SUV was 8.08 (Fig. 2). Pathological examination was performed on the tonsil mass and the results indicated adenocarcinoma. Immunohistochemical staining showed CDX-2(−), CK20(+), CK7(−) and TTF-1(−) in the tumor cells, confirming metastasis from colorectal adenocarcinoma (Fig. 1). The patient was then treated by chemotherapy with irinotecan, calcium folinate and fluorouracil every 14 days for four cycles; however, the patient succumbed to progression of the disease.

## Discussion

PET-CT scanning is important in oncology; functional and anatomical images are captured in a single scanning process. The PET-CT approach offers extensive possibilities for improving the diagnosis of primary and metastatic tumors (5). In the present case report, the patient denied any fever, pharyngalgia or night sweats. Thus, PET-CT was considered to be the best tool to identify insidious metastasis, improving radiotherapy planning and monitoring the effects of chemotherapy.

Identification of the primary tumor site is a challenging issue, which has prognostic and therapeutic significance. In the current case report, PET-CT revealed a mass in the left side of the tonsil. To identify the original site of the tumor, immunohistochemistry was performed and the results indicated a CK7(−)/CK20(+) phenotype, which confirmed colorectal carcinoma (6).

Malignant tumors seldom metastasize to the oral cavity. When this occurs, such metastasis is usually from various organs, including the lung, breast and rectum. According to previous studies, a few cases of metastatic localization have been identified from the colon and rectum, including the gingival, alveolar mucosa and tongue (7–9). The current case report presents a case of a 67-year-old male with tonsil metastasis from rectal adenocarcinoma. To the best of our knowledge, this is the first recorded instance of tonsil metastasis from rectal adenocarcinoma.

Since inflammatory and reactive lesions are common in the oral cavity, it is challenging to diagnose the rare case of rectal metastatic tumors in the tonsil. Occasionally, patients with tonsil metastasis clinically mimic patients with the primary tonsil neoplasm or tonsillitis. Therefore, it must be considered in the differential diagnosis of patients with unexplained pharyngalgia and antiadoncus. Careful examination, as well as a multidisciplinary approach, is recommended to validate the clinical suspicion. In conclusion, the current case report presents a unique case of tonsil metastasis from rectal adenocarcinoma and the oncologist must recognize the possibility of tonsil metastasis from malignant disease, such as colorectal cancer.
